# Different mechanisms for human rhinovirus survival in the presence of deleterious amino acid substitutions at virion protein-protein or RNA-protein interfaces

**DOI:** 10.1128/jvi.00511-26

**Published:** 2026-06-10

**Authors:** Juan Carlos Gil-Redondo, Valentín Riomoros-Barahona, Luis Valiente, Alejandro Valbuena, Mauricio G. Mateu

**Affiliations:** 1 Centro de Biología Molecular Severo Ochoa (CSIC-UAM), Universidad Autónoma de Madrid 16722, Madrid, Spain; The University of Texas Southwestern Medical Center, Dallas, Texas, USA

**Keywords:** virus, assembly, stability, uncoating, protein-protein interactions, RNA-protein interactions, compensatory mutations

## Abstract

A combination of structure-function and genetic analyses of viruses is unveiling a wide repertoire of adaptive mechanisms for restoring biological function in the face of negative selection pressures. The fixation of second-site compensatory mutations in highly heterogeneous RNA virus populations constitutes a frequent and versatile response to recover infectivity in the presence of deleterious mutations. In this study, we explored the following: (i) survival mechanisms of human rhinovirus (RV) when infectivity-impairing amino acid substitutions were introduced at either capsid protein-protein interfaces or capsid protein-RNA interfaces involved in virion assembly and viral RNA uncoating, and (ii) whether recovery of infectivity through compensatory amino acid substitutions in different functional elements in the same virion may be constrained to different extents. Specific interactions between capsid subunits or between the capsid and the genomic RNA in the RV virion were disrupted by replacement of individual amino acid residues with alanine, and the resulting crippled viruses were left to recover normal infectivity during serial infections of human cells. Identification of compensatory amino acid substitutions acquired during virus multiplication revealed that RV can use different genetic mechanisms to recover infectivity, depending on whether the deleterious substitution was localized at either pentamer-pentamer or capsid-RNA interfaces. Moreover, severe constraints to restore infectivity were found when deleterious substitutions were introduced at capsid-RNA interfaces instead of inter-pentamer interfaces. Because of those constraints, the capsid-RNA interface may constitute a preferential target for the design of anti-RV drugs that can interfere with viral assembly and uncoating, while minimizing opportunities for virus escape.

**IMPORTANCE:**

Human rhinoviruses cause most common colds and originate serious socioeconomic problems every year. In addition, they are associated with or exacerbate severe respiratory diseases, including chronic obstructive pulmonary disease, a major cause of death worldwide. No vaccines or drugs against rhinovirus infection are currently available. This study shows that when deleterious amino acid substitutions are introduced at interfaces between rhinovirus capsid subunits, infectivity is readily recovered through the fixation of second-site compensatory mutations that restore virion assembly and conformational stability. In contrast, severe difficulties were encountered for the recovery of infectivity when similar deleterious substitutions that impair virion assembly and stability and disregulate genome uncoating are introduced at capsid-viral RNA interfaces. The results suggest that the capsid-RNA interface may constitute a promising target for the design of anti-rhinoviral drugs that impair virus assembly and uncoating, while restraining the emergence of drug-resistant variant viruses.

## INTRODUCTION

Propagation of RNA viruses involves rapid replication, high mutation rates, and large population sizes, all of which give rise to extremely heterogeneous mixtures of genetic variants, termed viral quasispecies (1–3). Such a high genetic heterogeneity facilitates, during virus multiplication, the fixation in the viral population of pseudo-reversions or second-site compensatory mutations as a powerful adaptive response to the eventual occurrence of deleterious primary mutations (4–18).

Structure-function analysis of the genetic response of an RNA virus for survival in the presence of crippling mutations is unveiling multiple, sometimes complex mechanisms for restoring viral function when confronted with negative selection pressures (19–37). Of particular interest from an applied perspective is the genetic response of human immunodeficiency virus type 1 (HIV-1) and other viruses to the selection pressure exerted by antiviral drugs. Drug-escape mutations frequently impair virus infectivity and biological fitness; because fitness recovery through genetic reversion would also restore drug sensitivity, second-site compensatory mutations that restore fitness, but not drug sensitivity, are frequently selected for (38–52).

Rhinoviruses (RVs) are small, nonenveloped, single-stranded (ss) RNA viruses of the *Enterovirus* genus within the *Picornaviridae* family (53, 54). RVs constitute excellent models for both fundamental and applied research on highly variable RNA viruses. RVs are responsible for most common colds, which cause serious socioeconomic problems every year. They are also associated with, or exacerbate, severe respiratory diseases including asthma, pneumonia, and chronic obstructive pulmonary disease, the latter being a major cause of death worldwide (55–57). Pleconaril and other organic compounds that bind within large hydrophobic pockets in the capsid of RV-A or RV-B species showed great promise as anti-RV agents but failed clinical trials (58–60). Despite renewed efforts and substantial advances in anti-enteroviral research (61–65), no anti-RV or anti-enteroviral drug has been approved so far.

**Fig 1:**
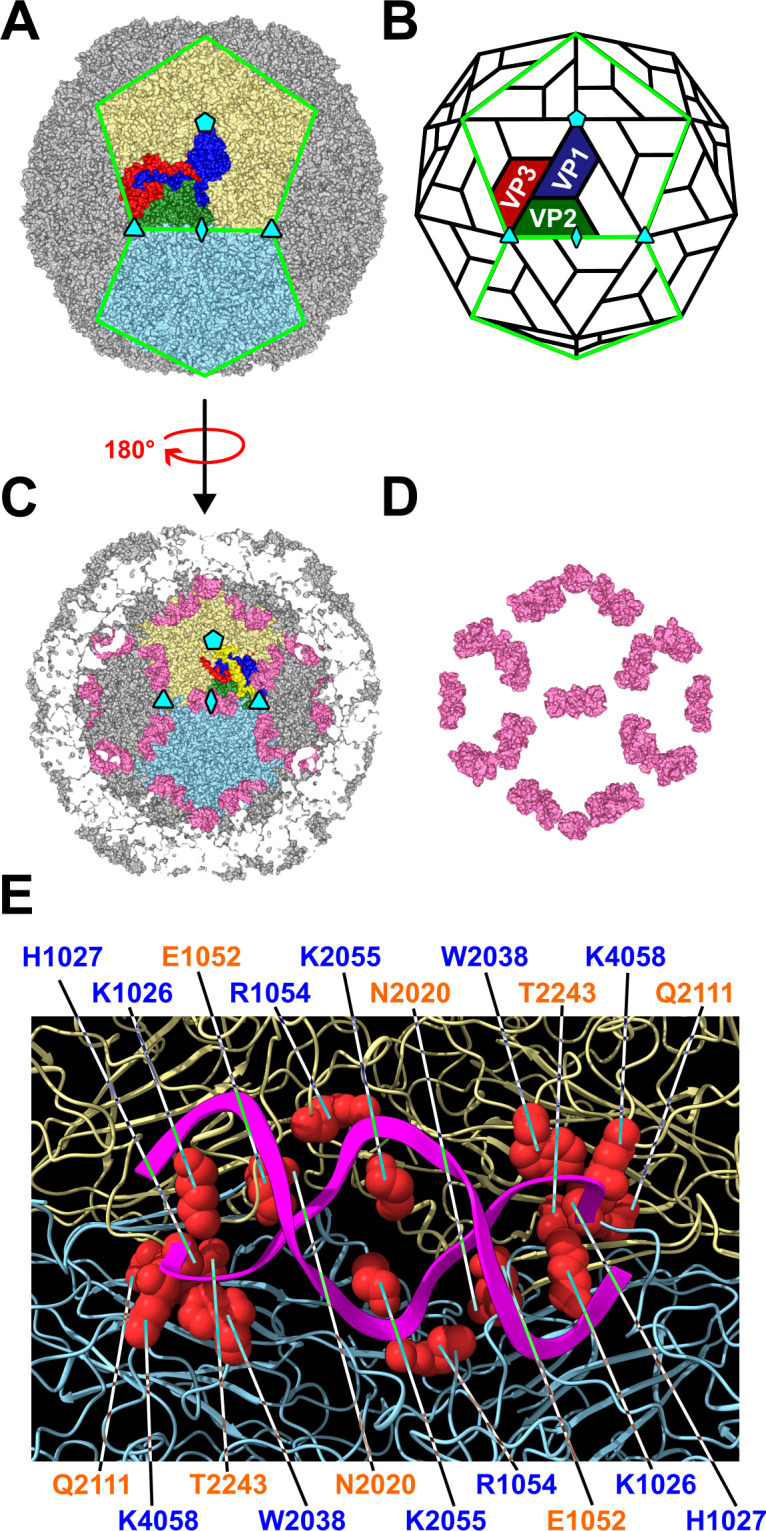
RV-B14 virion structure and amino acid residues at capsid pentamer-pentamer or capsid-duplex RNA interfaces that were subjected to deleterious replacements. (**A**) Atomic structure of the RV-B14 virion, as determined at a high resolution by cryo-EM (PDB ID: 8PNF) (67). (**B**) Schematic diagram of the icosahedral RV capsid. In panel A, two neighbor capsid pentamers are, respectively, colored light yellow and cyan. In both panels A and B, these two pentamers are delimited by green lines; one capsid protomer is colored blue (VP1), green (VP2), and red (VP3); and some icosahedral capsid 5-fold, 3-fold, and 2-fold symmetry axes are, respectively, indicated by cyan pentagonal, triangular, and rhomboidal symbols. (**C**) Cutaway view of the RV-B14 virion structure, rotated 180° around the vertical axis relative to the orientation represented in panel A. The same color code and symbols used in panel A to highlight two capsid pentamers, one capsid protomer, and some capsid icosahedral symmetry axes are used in panel C. The internal VP4 polypeptide belonging to the highlighted capsid protomer is colored deep yellow. The structured duplex RNA elements bound to the inner capsid wall (67) are colored magenta. (**D**) Organization of the 30 capsid-bound RNA duplexes in the shape of a dodecahedral cage inside the virion. (**E**) Close-up view of a pentamer-pentamer interface in the RV-B14 virion structure and a capsid-bound duplex RNA interface, as viewed from inside the virion. Parts of the structures of the two interacting pentamers are schematically represented using a ribbon model, and color-coded as in panel A, with an icosahedral 2-fold symmetry axis located at the center of the image. The RNA duplex is schematically represented using a magenta ribbon model. The capsid residues involved in pentamer-pentamer interactions or in capsid-duplex RNA interactions are represented as red space-filling models. Orange or blue labels identify capsid amino acid residues involved in pentamer-pentamer or capsid-RNA interactions, respectively.

The pseudo-T = 3 icosahedral picornavirus capsid (30 nm in diameter) is made of 60 copies of each of 3 capsid proteins (VP1, VP2, and VP3) and a shorter internal polypeptide (VP4) that may be considered a detached N-terminal extension of VP2(66)(Fig. 1A and B). The capsid encloses the viral ssRNA molecule (~7,200 nucleotide-long in RV). At least in the RV virion, the ssRNA molecule is organized as a dodecahedral “cage” delimited by 30 capsid-bound double-stranded (ds) RNA elements (67) (Fig. 1C and D). These well-structured, intra-chain RNA duplexes are specifically bound to concavities at the capsid inner wall located around 2-fold symmetry axes (Fig. 1E).

Picornavirus morphogenesis involves several steps (68). The capsid proteins are translated as a single polyprotein (P1). The folded P1 is proteolytically processed to yield a protomeric assembly intermediate that contains VP0 (VP2 with VP4 covalently attached to its N-terminus [Nt]), VP1, and VP3. Pentameric subunits are formed by association of five protomers, and an immature virion is built in a viral factory by co-assembly of 12 capsid pentamers and the genomic RNA molecule (69). In a final step, VP0 is internally processed into VP4 and VP2 to yield a mature, infectious virion (70).

Mature virions of the RV major receptor group, such as RV-B14 used in this study, specifically recognize protein ICAM-1 on the host cell surface. Binding to this cell receptor triggers both the internalization of the virion through the endosomal route and a conformational rearrangement of the viral particle that releases VP4 and externalizes the VP1 Nt. In a subsequent step, acidification at the endosome triggers another conformational rearrangement that facilitates uncoating of the RNA genome, which is translocated into the cytosol, where it is replicated and expressed (71–76). The viral RNA is released from poliovirus (77), and probably also from enterovirus 71 (78) and RV (79, 80), through one of the transient pores opened at the capsid 2-fold axes. These pores are located at the centers of the pentamer-pentamer interfaces, which are also the places where the structured RNA duplexes are bound (67) (Fig. 1E). Pleconaril and other capsid pocket-binding anti-RV compounds increase the virion mechanical stiffness (81) and impair the uncoating process (58–60, 82).

In previous studies on RV, we had found that in addition to capsid pocket-lining residues (35), many capsid residues involved in either pentamer-pentamer interactions or capsid-duplex RNA interactions play important roles in virion assembly and/or the modulation of viral genome uncoating (83, 84). Because of their dual functional role in RV morphogenesis and genome uncoating modulation, both the inter-pentamer interfaces and the capsid-duplex RNA interfaces may constitute promising new targets for the development of antiviral drugs that interfere with those critical stages of the RV infectious cycle.

In the present study, we have used a compensatory mutation-based approach to investigate adaptive mechanisms for RV survival when deleterious amino acid substitutions were introduced at the biologically relevant inter-pentamer or capsid-duplex RNA interfaces in the virion. The results revealed that recovery of RV infectivity is subjected to different constraints and proceeds through different mechanisms, depending on whether the deleterious substitution is localized at pentamer-pentamer (inter-protein) interfaces or at capsid-RNA interfaces. More severe constraints may exist for restoring infectivity when deleterious substitutions occur at capsid-RNA interfaces than when similar deleterious substitutions occur at pentamer-pentamer interfaces. Implications for antiviral drug design are discussed.

## RESULTS

### Deleterious substitutions introduced at pentamer-pentamer or capsid-RNA interfaces in the RV virion for analysis of virus survival strategies based on compensatory mutations

Ten residues in the RV-B14 capsid involved in non-covalent interactions, either between capsid pentamers (83) or between the capsid and the organized RNA duplexes inside (84), were chosen for individual replacement by alanine (Table 1 and Fig. 1E). To minimize the possibility of genetic reversion during virus multiplication, two nucleotides in the mutated codon were mutated in each case. All of the introduced primary amino acid substitutions disrupted either pentamer-pentamer interactions or capsid-duplex RNA interactions (Table 2, left side) and were deleterious. Nine out of those 10 substitutions drastically reduced virus infectivity titers by ~3–4 orders of magnitude. The W2038A substitution was lethal, as no progeny mutant virus could be recovered (84) (Table 1). Thus, an additional mutant, W2038F, was also analyzed. Compared to W2038A, W2038F carries a sterically more conservative amino acid replacement (phenylalanine instead of alanine) of the tryptophan at position 38 of VP2. This latter substitution was not lethal, but it still reduced infectivity at 72 h post-transfection (p.t.), and impaired fitness relative to the non-mutated (wild-type, wt) virus in biological competition experiments (84).

**TABLE 1:** Deleterious amino acid substitutions introduced at pentamer-pentamer or capsid duplex RNA interfaces in the RV-B14 virion

Amino acid substitution^*a*^	Residues in RV-A,B^*b*^	Mutated codon^*c*^	Relative infectivity^*d*^	Assembly^*e*^	Stability^*f*^
No			1	+	+
Pentamer-pentamer interface					
E1052A	E (100%)	GAA→GCU	(2.1 ± 3.2) × 10^−4^	+	-
N2020A	D (62%), N (38%)	AAU→GCU	(1.7 ± 1.9) × 10^−3^	-	+
Q2111A	Q (100%)	CAG→GCG	(1.3 ± 0.9) × 10^−4^	-	-
T2243A	T (73%), S (27%)	ACA→GCU	(6.5 ± 7.2) × 10^−3^	-	+
Capsid-duplex RNA interface					
K1026A	T (29%), K (27%), S (22%), Q (6%), V (5%), H (5%), A (3%), N (2%), L (1%), I (1%)	AAA→GCA	(4.5 ± 7.2) × 10^−3^	+	-
H1027A	T (63%), H (24%), S (7%), Y (3%), I (3%)	CAC→GCC	(7.1 ± 1.8) × 10^−2^	-	-
R1054A	R (100%)	AGA→GCA	(1.5 ± 2.1) × 10^−4^	-	+
K2055A	Q (41%), R (24%), K (20%), H (15%)	AAA→GCA	(1.2 ± 0.2) × 10^−4^	-	+
K4058A	K (100%)	AAA→GCA	(9.2 ± 8.0) × 10^−3^	-	-
W2038A	W (100%)	UGG→GCG	(<1.7 ± 1.6) × 10^−6^	NT^*g*^	NT
W2038F		UGG→UUC	(1.4 ± 1.8) × 10^−1^	-	-

^*a*^ The first digit indicates the VP (VP1, VP2, or VP4), and the following three digits indicate the position of the residue in the protein.

^*b*^ Residues found at the corresponding position in 106 serotypes of RV species A or B, and (in parentheses) percent conservation.

^*c*^ Mutated codons. In every case, two nucleotide substitutions were introduced.

^*d*^ Relative infectious titer is expressed as (mutant infectious titer: wild-type infectious titer) ratio at 48 h p.t., except for W2038F, for which the titer at 72 h p.t. is indicated. Average values from independent experiments and standard deviations are indicated. Data taken from references 83 and 84.

^*e*^ Qualitative effects on virion assembly. +, no or insignificant effect; -, substantial to drastic effect (reduced virion assembly efficiency). The original numerical values obtained for most primary mutants were included in references 83 and 84. The assembly efficiency of several primary mutants was analyzed again in the present study, as described in Results, and the values obtained were consistent with the previously obtained values.

^*f*^ Qualitative effects on virion stability, which were inversely related to the genome uncoating rate (84). +, no or insignificant effect; -, substantial to drastic effect (reduced thermostability that tends to result in increased RNA release). The original numerical values obtained for most primary mutants were included in references 83 and 84. The stability of several primary mutants were analyzed again in the present study, as described in Results. The new results were consistent with the previous results, except for the stability of the N2020A mutant virus. Duplicate experiments revealed that the N2020A mutant virus stability is not significantly different from the wt control. Assembly of the E1052A mutant virus had not been previously assessed and was analyzed in the present study. This mutant yielded an amount of assembled virions (83% ± 16%) that was not significantly different from that obtained with the wt control.

^*g*^ NT, not testable, as the mutation was lethal and no virions were recovered.

**TABLE 2:** Net number of interactions lost (or gained) as a consequence of deleterious primary substitutions at either the inter-pentamer interface or the capsid-duplex RNA interface, and net number of interfacial interactions potentially gained (or lost) as a consequence of infectivity-restoring substitutions^*a*^

Deleterious primary substitution	Interactions lost (−) or gained (+)^*b*^			Infectivity-restoring substitution	Pentamer-pentamer interactions gained (+) or lost (−)^*c*^			Capsid-RNA interactions gained (+) or lost (−)^*c*^		
	vdW^*d*^	H-bond	Ionic		vdW	H-bond	Ionic	vdW	H-bond	Ionic
Pentamer-pentamer interface										
E1052A	−11 (−4)	−1	−1	T3164P	0	0	0	0	0	0
N2020A	−5 (0)	−2	0	S2068N	+10 (+1)	+2	0	0	0	0
Q2111A	−4 (−1)	−1	0	N3077H	+10 (+5)	+1	+2	0	0	0
				N3077K	+6 (+5)	0	+2	0	0	0
T2243A	0	−1	0	I3128M	+2 (+1)	0	0	0	0	0
				I2254V	−3 (−2)	0	0	0	0	0
Capsid-duplex RNA interface										
K1026A	0	0	−1	L2018F	-2	0	0	0	0	0
H1027A	0	0	−2	D2057E+T4039A	0	0	−1	0	0	0
				M3146G	−16 (−6)	0	0	0	0	0
R1054A	0	0	−2	T3164P	0	0	0	0	0	0
K2055A	0	0	−2	I3128L	0	0	0	0	0	0
K4058A	−14 (−6)	−1	−3	T2243S	0	−1	0	0	0	0
W2038A	−25 (−16)	0	0	No^*e*^	NA^*f*^	NA	NA	NA	NA	NA
W2038F	+1 (+1)	0	0	T2243A	0	−1	0	0	0	0

^*a*^ Pentamer-pentamer and capsid-RNA contact analysis was performed as described in Methods, using the RV-B14 refined crystal structure (PDB ID: 4RHV) (85) or a cryo-EM structure (PDB ID: 8PNF) (67), respectively. Cutoff distances: van der Waals (VdW) pairwise contacts, sum of the vdW radii of the two atoms considered ± 0.5 Å; hydrogen bond (H-bond), 3.5 Å between donor and acceptor atoms; opposite charge-charge (ionic) interactions: 10 Å between charged groups. For the infectivity-restoring substitutions, contact analysis was performed using the simplifying assumption that the substituted side chain adopted the energetically most favorable conformer, but that no other conformational rearrangement occurred in the virion as a consequence of that substitution.

^*b*^ Net number of interactions lost (−) or gained (+) as a consequence of the deleterious primary substitutions.

^*c*^ Net number of interactions gained (+) or lost (-) at the pentamer-pentamer interface or at the capsid-RNA interface as a consequence of the infectivity-restoring second-site substitutions.

^*d*^ Number of vdW contacts and (in parentheses) number of carbon-carbon vdW (“hydrophobic”) contacts.

^*e*^ No compensatory mutation was fixed in the whole P1 capsid region, and no recovery of infectivity was detected.

^*f*^ NA, not applicable.

The compromised infectivity of the 11 primary mutants included in the present study was traced, in our previous analyses (83, 84), to impaired virion assembly, and/or to reduced virion thermostability, which resulted in facilitated unproductive viral RNA release. For many of those mutant viruses (5 out of 11), both virion assembly and stability were impaired (Table 1). Recovery of infectivity could be difficult in those cases because functional deficiencies in not one, but two completely different stages of the infectious cycle should be restored. Simple genetic reversion was highly unlikely, as it would require the successive fixation of two different nucleotide substitutions in the mutated codon. Indeed, some of the chosen interfacial residues are highly conserved among strains of the closely related RV-A and RV-B species. However, some others are not conserved (Table 1), which suggests that second-site substitutions that compensated for a deleterious effect of primary substitutions at inter-pentamer or capsid-RNA interfaces may have occurred during circulation of RVs in nature.

Human cells were transfected as described in Methods with wt RV-B14 or mutant RV genomes obtained from recombinant clones containing the chosen primary, deleterious substitutions. For several mutants, independent duplicate transfections were performed to obtain two different starting virus populations. Those viral populations were used to serially infect fresh human cell monolayers. Progeny viruses eventually obtained from each round of infection were used to infect another fresh cell monolayer in blind passages, using the procedure described in a previous study (35). During this serial passaging of the viruses in cultured cells, the relative virus infectivity and the consensus sequence of the population were determined at least for (i) the initial virus population obtained by transfection; (ii) the virus population obtained after a number of serial passages until a clear cytopathic effect was observed (which indicated that infectivity had been at least partially recovered); and (iii) the virus population obtained after further serial passages that led to the domination or imposition of a compensatory mutation in the population.

### Second-site compensatory substitutions fixed in response to deleterious substitutions introduced at inter-pentamer interfaces in RV

We analyzed first the virus mutants that carried deleterious amino acid substitutions at the capsid inter-pentamer interfaces (Table 3).

**TABLE 3:** Virus variants with deleterious substitutions at pentamer-pentamer interfaces, and infectivity-restoring substitutions that occurred during serial infections

Deleterious amino acid substitution^*a*^	Series^*b*^	Passages to cpe^*c*^	Titer at cpe^*d*^	Passages to compensatory mutation^*e*^	Titer after compensatory mutation^*f*^	Infectivity-restoring substitution^*g*^	Mutated codon^*h*^
No		NA^*i*^	1.0*	NA	1.0*	No	No
E1052A		3	0.5 ± 0.1	3	0.5 ± 0.1	T3164P	**A**CA → **C**CA
N2020A		3	1.0 ± 0.2	5	8.8 ± 2.3	S2068N (60%)	A**G**U→ A**A**U
Q2111A	1	1	0.6 ± 0.2	2	0.5 ± 0.2	N3077H	**A**AU → **C**AU
Q2111A	2	2	5.0 ± 0.3	2	5.0 ± 0.3	N3077K	AA**U**→ AA**A**
T2243A	1	5	1.9 ± 0.1	5	1.9 ± 0.1	I2254V	**A**UA → **G**UA
T2243A	2	2	13.7 ± 4.7	6	11.9 ± 2.3	I3128M	AU**U**→ AU**G**

^*a*^ No indicates that no mutation was introduced in the viral genome; the reference values given (labeled with an asterisk) correspond to the wt (RV-B14) virus control.

^*b*^ For mutants Q2111A and T2243A, two independent series of infections (passages in cell culture) were performed (respectively denoted Ser 1 and 2), using as a starting point two different mutant virus populations.

^*c*^ Number of passages required for observing a clear cytopathic effect (cpe).

^*d*^ Infectivity titer of the virus recovered after a clear cpe was observed, relative to the wt control virus. The infectivity values are expressed as the ratio between the mutant infectious titer and the wt infectious titer obtained in the same experiment (* reference value). The titers were obtained in duplicate; the average value ± standard deviation is indicated in each case. The relative titers of the primary mutants before any serial passaging are indicated in Table 1; all of them were ~ 2-4 orders of magnitude lower than the wt reference titer.

^*e*^ Number of passages required until a compensatory mutation was imposed in the virus population and close to normal infectivity was recovered.

^*f*^ Infectivity titer of the virus recovered after a compensatory mutation was imposed in the population, relative to the wt control virus. The infectivity values are expressed and were obtained as indicated in footnote d.

^*g*^ Capsid amino acid substitutions fixed in the virus populations recovered after close to normal infectivity were recovered, relative to the original sequence (before serial passaging). These second-site substitutions were imposed in >95% of the viral population, except for S2068N that was present in most but not all viruses (60%) in the population. No silent mutations were detected in any case.

^*h*^ The original and mutated codons that resulted in amino acid substitution(s) during the serial passages are also indicated. The mutated nucleotides are indicated in boldface. No mutations were detected in the wt virus when it was repeatedly subjected to >10 serial passages under the same conditions used for the mutant viruses.

^*i*^ NA, not applicable.

The results showed that in most cases, as few as 1–3 serial passages were enough for the virus to recover wt-like infectivity (relative infectivity >0.5). The only exception involved one of the two serial passages of mutant virus T2243A, in which extensive cytopathic effect and infectivity recovery required five passages. However, even for T2243A, a high number of passages was not necessary for restoring infectivity, as in the other series of infections using this same mutant, only two passages were required.

The entire genomic P1 region of the virus population obtained with each mutant at the end of each serial infection process was sequenced. The results revealed that the virus invariably responded to the introduction of a deleterious amino acid substitution at the inter-pentamer interfaces by fixation in the viral population of a second-site, compensatory amino acid substitution. For all but one mutant, >95% of the viruses in the final population had acquired a compensatory substitution. For mutant virus N2020A, the second-site substitution was not completely imposed even after five serial passages, but it did become dominant in the population (it was present in 60% of the progeny viral genomes). In every case, the second-site amino acid substitution resulted from a single-nucleotide change. None of the analyzed cases involved compensation by either genetic reversion (which would have required the unlikely fixation of two nucleotide changes) or pseudo-reversion. When two independent serial passages were performed with the same mutant, different second-site compensatory substitutions were found, involving different amino acids at either the same position or at different positions (Table 3). No additional mutations were detected in the entire capsid-coding region of the progeny population genome.

### Restoration by second-site compensatory substitutions of functional deficiencies in RV caused by deleterious substitutions at inter-pentamer interfaces

Three (N2020A, Q2111A, and T2243A) of the four virus mutants tested with deleterious amino acid substitutions at the inter-pentamer interfaces were deficient in virion assembly (83). In addition, two (E1052A and Q2111A) of those four virus mutants showed reduced thermostability (83), which is associated with facilitated genome release (84). We now asked whether the second-site amino acid substitutions fixed in the population restored normal virus assembly and/or thermostability, or their infectivity-restoring effect was due to alteration of other virus phenotypic traits/functions during the infectious cycle.

**Fig 2:**
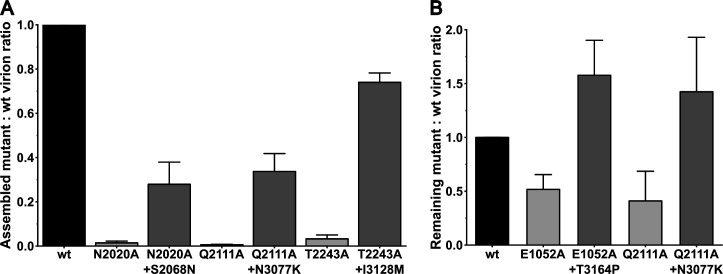
Effects on virion assembly efficiency and thermostability of deleterious primary amino acid substitutions, and infectivity-restoring amino acid substitutions at pentamer-pentamer interfaces in the virion. (**A**) Relative assembly efficiency and (**B**) relative thermostability of RV-B14 wt (black bar) and mutant virions carrying deleterious primary substitutions at pentamer-pentamer interfaces, either alone (light gray bars) or accompanied by infectivity-restoring second-site substitutions (dark gray bars). Error bars are indicated.

For each tested mutant and the wt control, virion assembly efficiency was determined by transfection of host cells with the viral genomic RNA as described previously (35). The RNA used for transfection was sequenced and found to contain both the primary mutation and the second-site mutation detected in the compensatory mutant obtained in the corresponding serial infection experiment. Virions were metabolically labeled with (35) S-Met/Cys, and the amount of virions assembled in a single infectious cycle was determined by ultracentrifugation and quantification of the radioactivity in the 150S peak, which corresponded to virions. The value obtained for each mutant was normalized by dividing it by the value obtained for the wt virus control in the same experiment. The normalized values obtained in two independent experiments were averaged. The results (Fig. 2A) confirmed that the deleterious, primary N2020A, Q2111A, and T2243A substitutions drastically reduced virus assembly efficiency. They showed that also for these three virus mutants, the second-site compensatory substitutions fixed in the corresponding viral population after serial passaging restored virion assembly efficiency to close to wt levels.

Likewise, for each tested mutant virus and the wt control, virion thermostability was determined by heating virus preparations at a defined temperature (45°C), and quantifying the reduction in infectious titer at different incubation times (0, 45, and 120 min.). The value obtained for each mutant was normalized by dividing it by the corresponding value obtained for the wt virus control in the same experiment. The normalized values obtained in two or three independent experiments were averaged. The results obtained using an incubation time of 45 min are indicated in Fig. 2B. These results confirmed that the deleterious, primary E1052A and Q2111A substitutions reduced virion thermostability. In addition, they showed that the infectivity-recovering second-site substitutions fixed in the corresponding viral populations obtained after serial passaging fully restored thermostability to wt levels. Analysis of the results at 120 min was consistent with the results obtained at 45 min. Because of the relationship found between infectious virion thermostability and viral genome uncoating control (84), these results indicated that the compensatory mutants were also likely to have regained normal control of viral RNA release.

### Localization in the RV virion structure of second-site amino acid substitutions that compensate for the deleterious effects of primary substitutions at inter-pentamer interfaces

**Fig 3:**
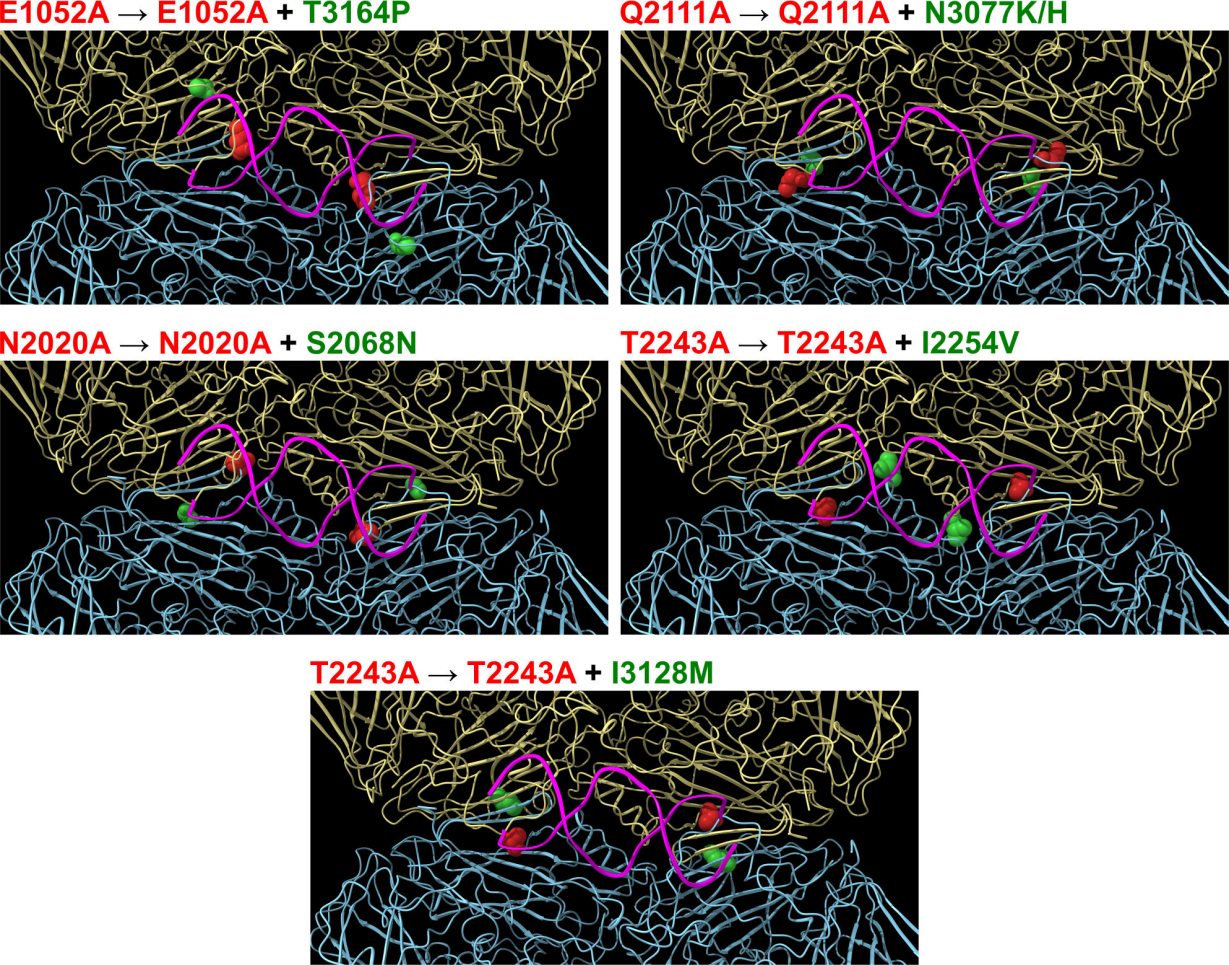
Localization of deleterious primary amino acid substitutions at pentamer-pentamer interfaces in the RV virion and of infectivity-restoring amino acid substitutions. Each panel represents the same close-up view of a pentamer-pentamer interface in the RV-B14 virion structure (67), as viewed from inside the virion. Parts of the structures of the two interacting pentamers are, respectively, represented using light yellow and cyan ribbon models. A capsid-bound RNA duplex element is represented using a magenta ribbon model. The capsid residues at the pentamer-pentamer interfaces whose primary replacement by alanine impaired viral infectivity are represented as red space filling models. The residues involved in infectivity-compensating substitutions that occurred during serial infections by infectivity-impaired virus mutants are represented as green space filling models. In each panel, the corresponding amino acid substitution in the original infectivity-impaired mutant is labeled in red color, and the second-site amino acid substitution that restored infectivity during the serial infections is labeled in green color.

For each deleterious primary amino acid substitution, the second-site amino acid substitution(s) that restored normal virus infectivity by restoring normal virion assembly and/or thermostability was mapped in the RV-B14 atomic structure. The results (Fig. 3) showed that all of the second-site compensatory substitutions occurred at or very near the capsid inter-pentamer interfaces, and fairly close to the corresponding originally substituted amino acid residue.

Analysis of the interactions lost (or gained) as a consequence of the primary replacement by alanine or potentially gained (or lost) because of the second-site compensatory substitution was performed using the non-mutated RV-B14 atomic structure (Table 2). As a starting, simplifying assumption, it was considered that the substituted side chain adopted the energetically most favorable conformer, but that no additional conformational rearrangements occurred in the virion as a consequence of that substitution.

The qualitative analysis suggested that in most cases, the inter-pentamer non-covalent interactions lost upon the introduction of deleterious primary substitutions (N2020A, Q2111A [two passage series], and T2243A [one series]) could be energetically compensated by new inter-pentamer interactions established nearby by the second-site substituted amino acid residue. For example, the primary Q2111A substitution would involve the loss of some pentamer-pentamer binding energy through the removal of a hydrogen bond and several van der Waals contacts. This loss could be compensated as a consequence of second-site N3077H or N3077K substitutions through the establishment of one additional inter-pentamer hydrogen bond and/or two ionic interactions, as well as several additional van der Waals contacts. In only two (E1052A and T2243A [one series]) out of six cases, additional pentamer-pentamer interactions were not predicted to be established as a consequence of the compensatory substitution (see the Discussion).

### Reversions and second-site compensatory substitutions fixed in response to deleterious substitutions introduced at capsid-duplex RNA interfaces in RV

We then analyzed the virus mutants that carried deleterious amino acid substitutions at the capsid-RNA duplex interfaces (Table 4).

**TABLE 4:** Virus variants with deleterious substitutions at capsid-duplex RNA interfaces and infectivity-restoring substitutions fixed during serial infections

Deleterious amino acid substitution^*a*^	Series^*b*^	Passages to cpe^*c*^	Titer at cpe^*d*^	Passages to compensatory mutation^*e*^	Titer after compensatory mutation^*f*^	Infectivity-restoring substitution^*g*^	Mutated codon(s)^*h*^
No		NA^*i*^	1.0*	NA	1.0*	No	No
K1026A	1	3	1.5 ± 0.1	3	1.5 ± 0.1	A1026K (rev)	**GC**A → **AA**A
K1026A	2	5	0.1 ± 0.1	9	0.1 ± 0.1	L2018F (80%)	**C**UC → **U**UC
H1027A	1	3	0.3 ± 0.1	12	0.9 ± 0.1	A1027H (rev, 60%) + M3146G (60%)	**GC**C → **CA**C **AT**G → **GG**G
H1027A	2	6	0.2 ± 0.1	13	1.0 ± 0.1	D2057E (80%) + T4039A (60%)	**AC**A → **GC**A GA**C** → GA**A**
R1054A		2	0.6 ± 0.1	2	0.6 ± 0.1	T3164P	**A**CA → **C**CA
K2055A		2	0.4 ± 0.1	4	0.5 ± 0.1	I3128L (75%)	**A**UU → **C**UU
K4058A	1	3	0.8 ± 0.2	3	0.8 ± 0.2	A4058K (rev)	**GC**A → **AA**A
K4058A	2	2	0.2 ± 0.1	8	1.3 ± 0.2	T2243S (85%)	**A**CA → **U**CA
							
W2038A	1	NA	NA	9	(9 ± 4) × 10^−6^	No	No
W2038A	2	NA	NA	10	<5 × 10^−7^	No	No
W2038F		0	1.9 ± 0.1	14	0.9 ± 0.1	T2243A (80%)	**A**CA → **G**CA

^*a*^ No indicates that no mutation was introduced in the viral genome; the reference values given (labeled with an asterisk) correspond to the wt (RV-B14) virus control.

^*b*^ For virus mutants K1026A, H1027A, K4058A, and W2038A, two independent series of infections (passages in cell culture) were performed (respectively denoted Ser 1 and Ser 2), using as a starting point two mutant virus populations.

^*c*^ Number of passages required for observing a clear CPE.

^*d*^ Infectivity titer of the virus recovered after a clear cpe was observed, relative to that of the wt control virus. The infectivity values are expressed as the ratio between the mutant infectious titer and the wt infectious titer obtained in the same experiment (* reference value). The relative titers were obtained in duplicate; the average value ± standard deviation is indicated in each case. The relative titers of the primary mutants before any serial passaging are indicated in Table 1; all of them were ~ 2–4 orders of magnitude lower than the wt reference titer, with two exceptions: W2038A was virtually lethal; W2038F titer was reduced by ~ 1 order of magnitude (at 72 h post-transfection).

^*e*^ Number of passages required until a compensatory mutation was imposed in the virus population and close to normal infectivity was recovered.

^*f*^ Infectivity titer of the virus recovered after a compensatory mutation was imposed in the population, relative to the wt control virus. For mutant virus W2038A, no compensatory mutation was acquired after the indicated passages; the titer obtained after the indicated blind passages is given. The infectivity values were obtained and are expressed as indicated in the previous footnote.

^*g*^ Amino acid substitutions fixed in the capsid of the virus recovered after close to normal infectivity were recovered, relative to the original sequence (before serial passaging). These second-site substitutions were imposed in > 95% of the viral population, except where indicated by a different percent value (in parenthesis). For example, substitution L2018F was fixed in ~ 80% of the population. For mutant virus W2038A, no compensatory substitution was acquired. No silent mutations were detected in any case.

^*h*^ The original and mutated codons that resulted in amino acid substitutions during the serial passages are also indicated. The mutated nucleotides are indicated in boldface. No mutations were detected in the wt virus when it was repeatedly subjected to > 10 serial passages under the same conditions used for the mutant viruses.

^*i*^ NA, not applicable.

The response of the virus when confronted with deleterious amino acid substitutions at the capsid-duplex RNA interfaces was different, more varied, and more complex overall than when confronted with deleterious substitutions at the inter-pentamer interfaces. As many as four different outcomes (numbered 1–4 below) were observed when alanine substituted any of the basic residues that surround each RNA duplex or the W2038 residue (which establishes many capsid-RNA contacts and exerts a strong hydrophobic effect by its stacking with each of the unpaired purines at the 5′ ends of each RNA duplex) (Table 4).

1. A second-site amino acid substitution that required a single nucleotide change only and that, like generally observed and described above for RVs with deleterious substitutions at the inter-pentamer interfaces, was imposed, or became dominant in the population, after only a few serial passages. However, compared to that observed for the inter-pentamer interface, in the capsid-RNA interface this outcome was extremely infrequent (R1054A and K2055A, only two out of 11 cases analyzed). Moreover, in one of these two cases, the second-site substitution (K2055A) had not been fully imposed even after four serial passages, whereas second-site substitutions at the inter-pentamer interfaces were generally fully imposed after 1–3 passages.
2. Second-site amino acid substitutions that required one nucleotide change only but that, unlike the second-site substitutions that occurred at the inter-pentamer interfaces, were not fully imposed even after many serial infections (8–14 passages, depending on the mutant and passage series). This response was frequently observed (5 out of 11 cases: K1026A [Ser. 2], H1027A [Ser. 1 and Ser. 2], K4058 [Ser. 2], and W2038F). For example, serial passaging of the W2038F mutant virus led to a quite slow and gradual imposition of the compensatory substitution T2243A, which in passages 3, 5, 7, 11, and 14 was, respectively, present in 0%, 25%, 65%, 75%, and 80% of the viral population. Moreover, in some cases, where compensatory substitutions were only gradually and not fully imposed, individual viruses in the same population recovered function through different second-site substitutions (H1027A Series 2), or alternatively, only a fraction of the viruses in a population recovered function through a second-site substitution, whereas another fraction in the same population recovered infectivity through a pair of nucleotide changes in the primary mutated codon that led to genetic reversion (H1027A Series 1).
3. Genetic reversion that restored the original, positively charged amino acid residue. This response was repeatedly observed (3 out of 11 cases; K1026A [Series 1], H1027A [Series 1], and K4058A [Series 1]). In the H1027A case, only half the progeny virus population had reverted; the other half of the population acquired a second-site compensatory substitution instead (see point 2). This recurrent response through genetic reversion is not trivial, as it requires in every case the improbable fixation of not one, but two nucleotide changes in the original codon.
4. No compensation at all. In the two independent serial passages performed, the mutant virus W2038A was unable, even after 9 (Series 1) or 10 (Series 2) passages, to revert, pseudo-revert, or find a second-site mutation that could rescue infectivity.

### Restoration by second-site compensatory substitutions or genetic reversion of functional deficiencies in RV caused by deleterious substitutions at capsid-duplex RNA interfaces

Two out of the six primary mutants tested with deleterious amino acid substitutions at the capsid-RNA interfaces were deficient in virion assembly but showed normal virion thermostability and genome uncoating; one other assembled normally but showed reduced stability and faster genome uncoating; and the remaining three showed impaired assembly, low stability, and faster uncoating (84) (Table 1). The same experimental approaches described above to analyze the effects of substitutions at the inter-pentamer interfaces were used now to analyze whether the recovery of infectivity of viruses with substitutions at capsid-RNA interfaces was due to restoration of virion assembly and/or thermostability.

**Fig 4:**
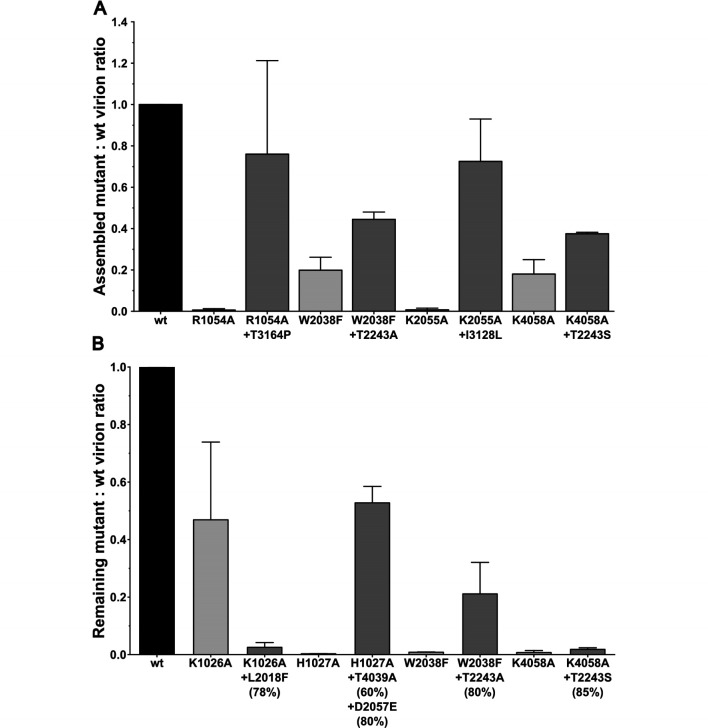
Effects on virion assembly efficiency and thermostability of deleterious primary amino acid substitutions, and infectivity-restoring amino acid substitutions at capsid-duplex RNA interfaces in the virion. (**A**) Relative assembly efficiency and (**B**) relative thermostability of RV-B14 wt (black bar) and mutant virions carrying deleterious primary substitutions at capsid-duplex RNA interfaces, either alone (light gray bars) or accompanied by infectivity-restoring second-site substitutions (dark gray bars). Error bars are indicated.

The results of the virion assembly analysis (Fig. 4A) revealed that the substantially or drastically reduced assembly efficiency of the R1054A, K2055A, W2038F, and K4058A mutant viruses was restored to close to wt levels through the corresponding second-site substitutions fixed after serial infections.

The results of the virion thermostability analysis (Fig. 4B) confirmed that the deleterious, primary H1027A, K4058A, and W2038F substitutions did substantially or severely reduce virion thermostability. They also showed that for the primary substitutions H1027A and W2038F, the infectivity-compensating substitutions restored thermostability to close to normal (wt) levels. However, the infectivity-compensating second-site substitutions did not restore the thermostability lost because of the K4058A primary substitution, or increased the moderate destabilization caused by the K1026A primary substitution. The entire capsid-coding region had been sequenced, and no other mutation was found.

### Localization in the RV virion structure of amino acid substitutions that compensate for the deleterious effects of primary substitutions at capsid-duplex RNA interfaces

**Fig 5:**
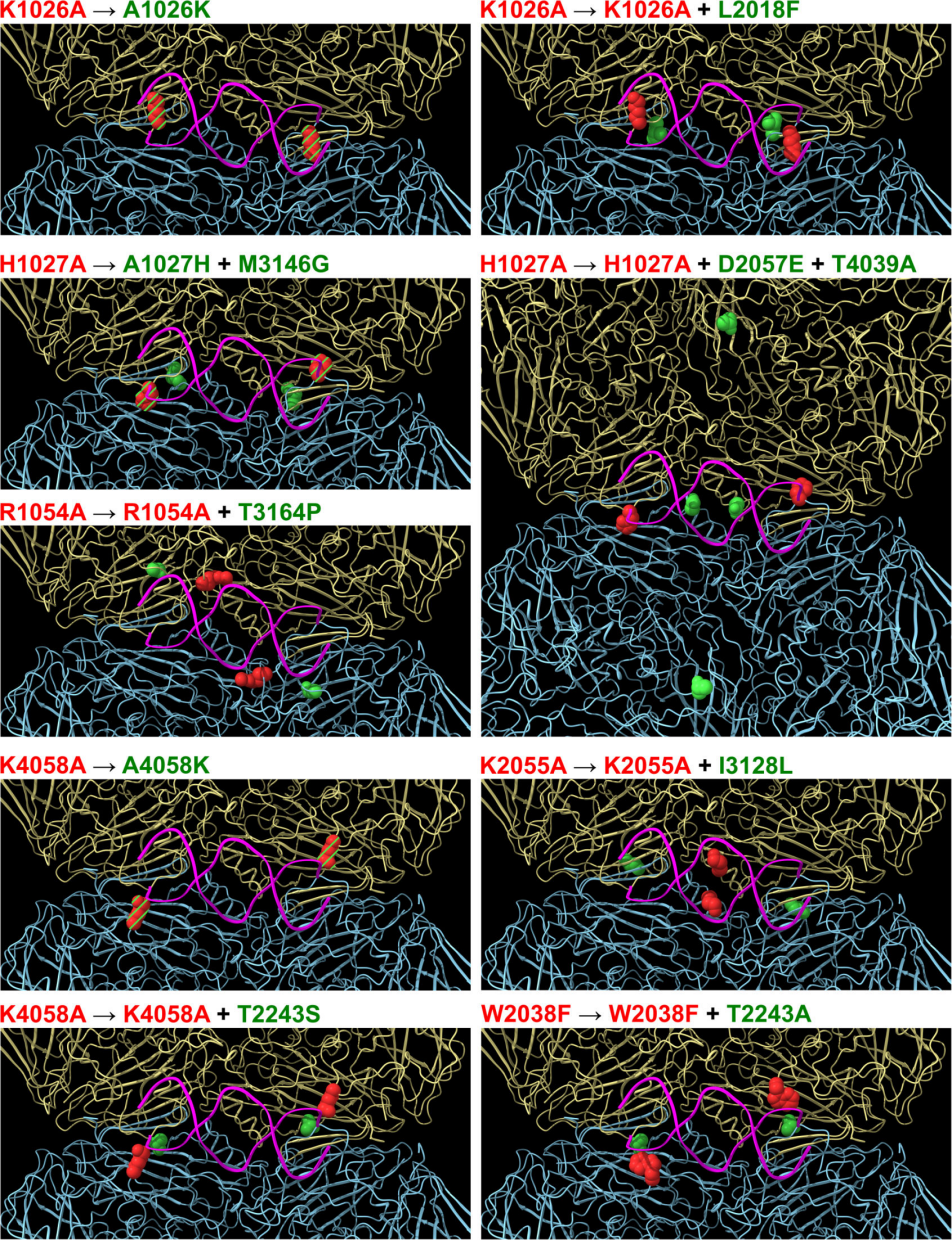
Localization of deleterious primary amino acid substitutions at capsid-duplex RNA interfaces in the RV virion, and of infectivity-restoring amino acid substitutions. Each panel represents the same close-up view of a pentamer-pentamer interface in the RV-B14 virion structure (67), as viewed from inside the virion. Parts of the structures of the two interacting pentamers are, respectively, represented using light yellow and cyan ribbon models. A capsid-bound RNA duplex element is represented using a magenta ribbon model. The capsid residues interacting with the RNA whose primary replacement by alanine impaired viral infectivity are represented as red space filling models. The capsid residues where infectivity-compensating substitutions occurred during serial infections with the infectivity-impaired virus mutants are represented as green space filling models. In the larger panel, the green residue farthest from the interface is T4039. In each panel, the corresponding amino acid substitution in the original infectivity-impaired mutant is labeled in red color, and the second-site amino acid substitution that restored viral infectivity is labeled in green color. In cases where a pseudo-reversion of the originally mutated residue occurred during serial infections, that residue is represented with a red/green bar pattern.

For each primary amino acid substitution tested at the capsid-RNA duplex interface, the compensatory substitution(s) that restored virus infectivity were mapped in the RV-B14 atomic structure (Fig. 5).

The results revealed that all but one (T4039A) of the compensatory substitutions occurred at or very near the capsid-duplex RNA interfaces. Most of them were also fairly close to the primary, deleterious amino acid substitution, with the exceptions of D2057E and I3128L, which, respectively, compensated for the deleterious effects of H1027A and K2055A. Most of the infectivity-restoring substitutions were also fairly close to the inter-pentamer interfaces (Table 4 and Fig. 5).

The deleterious K1026A substitution was compensated by a L2018F substitution at a spatially close position (Fig. 5, top right). It is interesting to note that in 3% of the RV-A, B serotypes analyzed, an alanine is found at position 1026 (Table 1) and that, in those three cases, an arginine is found at the position 2018. In these viruses, the lack of the positive charge at residue 1026 could have been compensated during virus circulation in the field by the introduction of a positive charge at the nearby residue 2018.

The interactions lost or gained at the capsid-RNA interfaces were analyzed, as for the inter-pentamer interfaces, under the simplifying assumption that the substituted side chain adopted the energetically most favorable conformer, but no additional conformational rearrangements occurred in the virion as a consequence of that substitution (Table 2). The deleterious primary substitutions analyzed removed either some coulombic interactions between positively charged amino acid residues and negatively charged RNA phosphates, or amino acid side chain-nucleotide base stacking interactions, both of which could reduce the capsid-RNA binding energy. The binding energy lost in each one of those cases does not appear to be recovered through new capsid-RNA interactions established by the infectivity-restoring second-site substitutions. We considered the possibility that at least some of those second-site substitutions, which are also close to the capsid pentamer-pentamer interface, could exert their compensatory effect by establishing new interactions between pentamers. However, inter-pentamer contact analysis under the same assumption stated above (Table 2) indicated that no such interactions occurred in any of those virus mutants.

## DISCUSSION

Viruses can acquire second-site mutations that compensate for the infectivity-impairing effects of other mutations, including drug-escape mutations. In this latter case, second-site mutations allow the recovery of normal infectivity and fitness without reacquiring drug-sensitivity. Given the extreme genetic heterogeneity of viral quasispecies, it could be thought that when any deleterious mutation whose reversion is disfavored is acquired by an RNA virus, a second-site mutation that would restore full infectivity and fitness could always be readily selected.

If the above view is correct without qualification, the discovery or design of target-specific antiviral drugs that RNA viruses cannot escape from could face serious difficulties. It should be considered, however, that mechanistically complex processes during the viral infectious cycle may be subjected to severe constraints. If a highly constrained viral function is compromised by a drug-escape substitution, appropriate second-site substitutions that can readily restore infectivity and fitness cannot not be readily found. If identified, highly constrained functional elements in pathogenic viruses may constitute preferential targets for antiviral drug design.

The present study has investigated the possibility that some functional elements of a pathogenic RNA virus may be under particularly stringent constraints for biological recovery through fixation of second-site mutations. The two functional elements of the RV virion chosen for a direct comparison here were the capsid pentamer-pentamer interface and the capsid-duplex RNA interface. Our previous structure-function analysis revealed that many amino acid residues at either interface play a critical role in virion assembly and/or thermostability (83, 84), the latter being required for controlled uncoating of the viral RNA genome (84).

The results obtained for the capsid pentamer-pentamer interface revealed that, during multiplication in host cells, second-site compensatory substitutions can be rapidly and readily fixed in response to deleterious substitutions that remove some inter-pentamer interactions. In every case, the second-site substitution involved one nucleotide change only, was fully imposed in the population, and completely restored normal infectivity in as few as 1–3 serial infections. It must be noted that, in three out of six cases, infectivity titers 5-fold to 10-fold higher than the wt were obtained. Significantly increased titers were never observed when the capsid-RNA interface was analyzed. One possibility to be considered is that, in rare cases, one additional mutation that could be fixed outside the capsid region could increase the virus replication efficiency in cultured cells.

The results obtained for the capsid-duplex RNA interface were quite different. Second-site substitutions were acquired in response to some, but not all, primary deleterious substitutions introduced at this interface. Moreover, a dominant presence in the viral population of most of those second-site substitutions required as many as 8–14 serial infections; even after all those passages, they were not fully imposed (they reached ~60%–85% only).

Assembly efficiency and stability were also not fully recovered by the viral progeny populations, which appear to consist of a mixture of viruses, ~60%–85% of them carrying the compensatory mutation. If so, infectivity titers of those populations should be expected to be ~60%–85% of the wt titer. This may well be the case, but the low accuracy of plaque assays does not allow for distinguishing between less than 2-fold differences in infectious titer.

In a number of cases (K1026A, H1027A, and K4058A), normal infectivity was recovered by restoring the original amino acid through genetic reversion, although this outcome involved the improbable fixation of not one, but two nucleotide changes in the corresponding codon. The situation was even more complex, as a heterogeneous mixture of two partially imposed second-site substitutions, or of a partially imposed second-site substitution and a reversion, was found after serial passaging of the H1027A mutant. Finally, in one case (W2038A), infectivity could not be recovered either through fixation of a second-site substitution or by reversion or pseudo-reversion, even after as many as ~10 blind passages in cell culture.

The above comparison indicates that, for RV, more severe constraints may exist for recovery from deleterious structural alterations at the capsid-duplex RNA interface than at the capsid pentamer-pentamer interface. This situation may not be trivially explained, as previous evidence and results of the present study, summarized next, support a structural and functional connection between both interfaces.

- The capsid-duplex RNA interfaces and the pentamer-pentamer interfaces run parallel and are located very close to each other at the capsid inner wall (Fig. 1E).
- A few capsid residues appear to be involved in both inter-pentamer interactions and capsid-RNA interactions (83, 84).
- Some secondary substitutions that compensated for the deleterious effects of primary substitutions at the capsid-RNA interface involved capsid residues located at or very close to the inter-pentamer interfaces (Fig. 5).
- In some cases, the same amino acid substitution, or different substitutions of the same residue, compensated for the deleterious effects of a substitution at the inter-pentamer interface and of another substitution at the capsid RNA interface. T3164P compensated for the deleterious effects of both E1052A at the inter-pentamer interface (Table 3) and R1054A at the capsid-RNA interface (Table 4); those three residue positions are close to each other in the RV capsid (Fig. 3 and 5). Likewise, I3128M compensated for the deleterious effect of T2243A at the inter-pentamer interface (Table 3), and I3128L compensated for the deleterious effect of R2055A at the capsid-RNA interface (Table 4); those three residue positions are also close to each other in the RV capsid (Fig. 3 and 5). In serial infections of viruses carrying a deleterious substitution at the capsid-RNA interface, T3164P and I3128L were the only second-site substitutions that became dominant after a limited number of passages. This outcome is similar to that observed for these two substitutions (and others) when they compensated for the deleterious effect of substitutions at the inter-pentamer interface.
- T2243A is in itself a deleterious substitution at the inter-pentamer interface (84). Remarkably, that same substitution, or another one at the same position, T2243S, respectively, compensated for the deleterious effects of substitutions W2038F and K4058A at the capsid-RNA interface (Table 4); residues T2243, W2038, and K4058 are located close to each other in the RV-B14 capsid (Fig. 5).
- Individual amino acid substitutions at either interface resulted in the same functional defect(s): impaired virion assembly and/or reduced virion thermostability leading to faster, uncontrolled RNA release (83, 84).
- Most (although not all) substitutions that compensated for the deleterious effects of primary substitutions at the capsid-duplex RNA interface or at the capsid pentamer-pentamer interface acted in the same way: they directly restored normal virion assembly efficiency and/or thermostability (and hence, controlled RNA release) (Fig. 2 and 4). In nearly every case, the compensatory substitution involved a capsid residue located at or fairly close to those interfaces and also fairly close to the originally substituted residue (Fig. 3 and 5).

Observations i to vii together provide strong support for a structural and functional linkage between the capsid inter-pentamer interface and the capsid-duplex RNA interface in the RV virion. Thus, one may ask why the mechanisms behind the functional restoration of virus infectivity were different when either interface was targeted: the deleterious effects of substitutions at the inter-pentamer interface were readily compensated by the rapid and complete fixation of second-site substitutions. In contrast, the deleterious effects of substitutions at the capsid-duplex RNA interface were generally reverted only by either the slow and partial imposition of second-site substitutions, or the improbable reversion to the original residue through two nucleotide changes, or, for the lethal W2038A substitution, the deleterious effect was not reverted at all.

Some observations provide initial support for a tentative hypothesis to explain the convoluted genetic response to deleterious substitutions at the capsid-duplex RNA interface, compared to the fast and straightforward response to deleterious substitutions at the pentamer-pentamer interface.

- The deleterious effect of every tested substitution at the inter-pentamer interface was compensated by restoring normal virion assembly and/or stability. However, the deleterious effect of a few substitutions at the capsid-RNA interface (K4058A and K1026A) that affected virion stability was not compensated for by restoring normal stability, but by another mechanism yet to be determined.
- Most (4 out of 6) second-site substitutions that compensated for the deleterious effect of substitutions at the pentamer-pentamer interface were predicted to establish additional inter-pentamer interactions. In contrast, none of the eight second-site substitutions that compensated for the deleterious effect of mutations at the capsid-RNA interface were predicted to introduce additional interactions, either between the capsid and the RNA duplexes or between the capsid pentameric subunits.
- Different experimental evidence has shown that the RV virion fluctuates between different similar conformations at equilibrium with each other (67). Interactions between the capsid and the RNA duplexes appear to control such conformational flexibility of the virion: substitutions that removed some of those interactions and that reduced infectivity by impairing virion assembly and/or stability actually led to changes in the conformational flexibility of the virion and, in particular, of some of their structural elements (J. R. Castón and M. G. Mateu, unpublished observations).

The above observations are consistent with the possibility that the deleterious effect of substitutions at the capsid pentamer-pentamer interfaces is generally due to the loss of some of the non-covalent interactions that hold the pentameric subunits together. A lower binding energy between the pentameric subunits would reduce virion assembly efficiency and/or stability (the latter leading to facilitated RNA release). In most (though not all) cases, establishing some additional non-covalent inter-pentamer interactions through second-site amino acid substitutions nearby would be enough to enthalpically restore normal virion assembly and stability.

In contrast, the deleterious effect of any substitution at the capsid-RNA interface could mainly depend on changes in virion conformational flexibility. As indicated in point (iii) above, deleterious substitutions at the capsid-RNA interface did alter the virion conformational dynamics. In one of the two serial passages of the E1052A or T2243A mutant viruses, additional pentamer-pentamer interactions were not predicted to be established when compensatory substitutions at inter-pentamer interfaces were acquired. As proposed for compensatory mutations at the nearby RNA-capsid interfaces, those two cases may also involve some changes in conformational flexibility. A testable prediction is that second-site substitutions that compensate for the deleterious effect of those primary substitutions would restore the conformational dynamics of the wt virion.

Second-site substitutions that can restore normal conformational dynamics (rather than compensate for lost interfacial interactions) could be rare in the viral quasispecies and provide a limited fitness increase only. If such were the case (under study), the virus would require many multiplication events before those substitutions could become dominant in the population, or it would resort to genetic reversion, as observed.

Whatever the structural basis for the observed constraints to recover viral infectivity, this study identifies the capsid-duplex RNA interface as a promising target for the design of novel anti-RV drugs. A small organic compound that could specifically bind a viral epitope containing capsid residues W2038 and K4058 could be a good drug candidate. Such a compound could include an aromatic moiety for stacking with W2038, and a negatively charged group for interaction with the positively charged K4058, plus a few other adequate moieties to increase affinity and specificity. Fitness recovery of the likely crippled escape mutants that could arise in response to this drug could be severely jeopardized because (i) this epitope has a critical dual function in two different stages of the infectious cycle, namely virion assembly and genome uncoating (84), and (ii) the present study has revealed that some substitutions of W2038 or K4058 that could lead to drug escape by preventing its binding could be lethal, and neither compensated nor reverted (W2038A); compensated, but only after many viral multiplication events (W2038F and K4058A); or reverted, which would lead to reacquisition of drug sensitivity (K4058A).

Although pleconaril and other drugs that bind the large hydrophobic pockets in the RV capsid failed clinical trials, new-generation anti-RV drugs that can bind those pockets are being considered. RV mutants resistant to pocket-binding compounds did arise in cell culture, but the effect of the escape mutations on virus biological fitness was not generally investigated (58–60). The evidence obtained with other drug-resistant mutations arising in different viruses supports the possibility that escape mutations in the RV pockets would actually reduce virus fitness and, thus, require the fixation of second-site mutations to restore normal fitness (38–52). In a very recent study, we used a compensatory mutation-based strategy to investigate possible structural constraints for the biological function of those druggable pockets in the RV capsid. Deleterious mutations leading to small-to-large substitutions of pocket-lining amino acid residues were introduced in the viral genome. The hampered viruses generally recovered normal infectivity not through the fixation of second-site mutations, but through same-site (pseudo-) reversions involving large-to-small amino acid substitutions that approximately restored the original pocket volume and shape. Further analysis revealed a connection between conservation of the pocket stereochemistry and preservation of the capsid flexibility needed for viral function (35).

Based on the severe constraints for acquisition by RV of second-site mutations that compensate for function-impairing mutations at those capsid pockets or at the capsid-duplex RNA interfaces, a combination therapy could be envisaged: targeting both structural elements (especially including the W2038/K4058 site discussed above) could result in a minimized generation of escape variants, despite the high genetic heterogeneity of this RNA virus.

To conclude, this study has revealed that during RV multiplication, second-site compensatory substitutions can be readily selected for. These substitutions restore virion assembly and/or stability-controlled viral RNA uncoating that were impaired by deleterious amino acid substitutions introduced at the inter-pentamer interfaces. In contrast, when deleterious substitutions were introduced at the capsid-RNA duplex interfaces, restoration of infectivity required more varied, complex, and less probable events: (i) genetic reversion (despite requiring two nucleotide changes instead of only one); (ii) one, or even two, non-ideal second-site substitution(s) that were only gradually and not fully imposed in the viral population. Moreover, for one particularly critical amino acid substitution at the capsid-RNA interface (W2038A), the virus was unable to recover infectivity at all. The results point to the capsid-RNA interface in the RV virion as a particularly promising target for the design of anti-RV drugs that can interfere with viral assembly and/or uncoating, while minimizing opportunities for virus escape.

## MATERIALS AND METHODS

### Infectious DNA clone and site-directed mutagenesis

The infectious recombinant DNA clone pWR3.26, encoding the complete RV-B14 viral genome, was obtained from the American Type Culture Collection (ATCC), amplified via transformation into *E. coli* XL1-Blue, and purified. Site-directed mutations in the capsid-coding region were introduced using the QuikChange II XL Site-Directed Mutagenesis Kit (Agilent) according to the manufacturer’s protocol. Each mutant plasmid was amplified through transformation into *E. coli* XL1-Blue and purified. Recombinant plasmids containing both a primary mutation and a second-site compensatory mutation were obtained by site-directed mutation on plasmids already containing the corresponding primary mutation, following the same procedures as stated above. The presence of the introduced mutation(s) and absence of other mutations were confirmed by automated sequencing of the full capsid-coding (P1) region of the RV genome (Macrogen and Plasmidsaurus).

### *In vitro* transcription of viral RNA

pWR3.26 recombinant plasmids containing the complete RV-B14 viral genome sequence, either wt or including the analyzed mutations, were linearized with MluI-HF (New England Biolabs) and purified by phenol extraction; 1 μg of linearized and purified DNA was transcribed into viral genomic RNA using 20 U of T7 RNA polymerase (Promega) in a transcription buffer containing 8 mM Tris-HCl (pH 7.9), 2 mM NaCl, 1.2 mM MgCl₂, 0.4 mM spermidine, 1% BSA, 10 mM DTT, 40 U RNasin, and 1 mM of each ribonucleoside triphosphate. The reaction mixture was incubated at 37°C for 2 h. The resulting RNA was briefly stored at −80°C for immediate use. Quantification of both linearized DNA and transcribed RNA was performed by agarose gel electrophoresis and spectrophotometry using a Nanodrop One spectrophotometer (Thermo Fisher Scientific).

### Culture of H1-HeLa cells and electroporation with RV-B14 RNA

HeLa-H1 cells, obtained from the ATCC, were cultured at 37°C in Dulbecco’s modified Eagle’s medium (DMEM) containing 2 mM glutamine, 100 U/mL penicillin, and 100 μg/mL streptomycin (DMEMc) and supplemented with 10% fetal bovine serum (FBS). For transfection, cells were electroporated with 15 μg of either wild-type or mutant RV-B14 RNA using a Gene Pulser II apparatus (Bio-Rad) under a single pulse (980 V, 25 μF, maximum resistance), as previously described (35). Negative controls were generated without RNA. Supernatants containing wt or mutant viral particles were harvested at 48 h post-transfection (following complete cytopathic effect in electroporated cells), centrifuged, and stored at −80°C for subsequent virus titration, serial infections, or nucleic acid sequencing.

### Serial infections of progeny virus populations for propagation and evolution

Confluent HeLa-H1 cells in 100 mm Petri dishes were washed with DMEMc, and the culture medium was aspirated. Cells were incubated with 600 μL of supernatant containing either wt or mutant viral progeny for 1 h. Following supernatant removal, the cells were maintained in DMEMc supplemented with 2% FBS at 35°C for 48 h. Supernatants were harvested, centrifuged, and stored at −80°C. This process was repeated serially for multiple passages using viral progeny from the preceding cycle, until a robust cytopathic effect comparable to that of wt virus populations was achieved. Additional passages were performed for selected mutants based on the progression of the cytopathic effect, as described in the Results section.

### Determination of infectious virus titers

Infectivity titers of viral populations produced after host cell transfection or serial infections were quantified in plaque assays. Nearly confluent HeLa-H1 monolayers in 60 mm Petri dishes were incubated for 1 h at 35°C with serial dilutions (in PBS) of viral suspensions, with each dilution tested in duplicate. In each assay, a wt RV-B14 control prepared under identical conditions was also included. After washing to remove unbound virus, the cells were covered with 0.7% agar in DMEM supplemented with 1% FBS and 0.5% DEAE Dextran, followed by incubation for 72 h at 35°C. Plaques were visualized after fixation in 2% formaldehyde and crystal violet staining. The mean plaque-forming units (PFUs) per mL from duplicate assays were normalized to the wt control to calculate relative infectivity values for each virus mutant.

### Extraction of RNA from viral populations in supernatants, reverse transcription, DNA amplification, and viral genome sequencing

Viral RNA from supernatants was isolated using TRIzol LS (Invitrogen) reagent following the manufacturer’s protocol and resuspended in DEPC-treated water (0.1% diethyl pyrocarbonate in H₂O). RNA concentration was quantified using a Nanodrop One spectrophotometer. The purified RNA was reverse transcribed and amplified via a RT-PCR protocol, employing specific primers (Custom Standard DNA Oligos, Thermo Fisher) targeting the capsid-coding P1 region, as previously described (35). RT-PCR products were visualized by agarose gel electrophoresis, purified using the Wizard SV Gel and PCR Clean-Up System (Promega), and quantified using a Nanodrop One. Final sequencing was performed by Macrogen or Plasmidsaurus. Nucleotide sequences of the entire P1 region were aligned and compared to the wild-type RV-B14 sequence using BioEdit v7.7.1. Mutations detected were confirmed through direct inspection of chromatograms using Chromas software (Technelysium Pty Ltd).

### Infectious virion thermostability analysis

The sensitivity of each mutant and wt RV-B14 virus to heat-induced infectivity loss was quantified as previously described (84). Briefly, 1.5 mL of viral suspensions (10⁴ PFU/mL) were incubated at 45°C in a temperature-calibrated thermoblock. Aliquots (0.5 mL) were collected at 0, 45, and 120 min of incubation and then titrated for infectivity via plaque assays as described above. Each dilution was tested in duplicate, and each assay included a wt virus control. Mean values from two independent experiments were obtained for each mutant.

### Virion assembly analysis

Viral assembly was analyzed for each mutant and wt RV-B14 control as previously described (84), with minor modifications. HeLa-H1 cells were transfected with viral RNA as described above, then cultured in Petri dishes with 2 mL of cysteine and methionine-free DMEM supplemented with 200 μCi [35S] methionine/cysteine (PerkinElmer) and 5% FBS. After 8 h at 35°C, the cell monolayers were lysed in NP-40 lysis buffer (10 mM Tris–HCl, pH 7.5, 10 mM NaCl, 1.5 mM MgCl₂, and 0.5% Nodinet P-40). Lysates were then layered on top of a sucrose cushion (30% wt/vol sucrose in PBS and 0.01% BSA) and centrifuged at 285,000 × *g* for 130 min at 4°C. Pellets were resuspended and subjected to ultracentrifugation in a linear sucrose gradient (7.5%–45% wt/vol sucrose in PBS, 0.01% BSA) at 285,000 × *g* for 110 min, without no braking; 0.45 mL fractions were collected from the resulting gradient, and the radioactivity present in them was quantified via liquid scintillation counting (RackBeta 1209, Wallac). Virion (150S) fractions were identified using calibrated sedimentation coefficient markers (thyroglobulin, minute virus of mice empty capsid, and RV-B14 virion). Radioactivity in each sample was normalized to that determined for the wt control virus. Mean values from two independent experiments were obtained for each mutant.

### Molecular graphics, image edition, and statistical analysis

Molecular graphics analyses were performed using UCSF Chimera (86) and the PDB files containing atomic coordinates for the refined crystal structure of the RV-B14 virion (PDB ID: 4RHV) (85) and the high-resolution cryo-electron microscopy of the RV-B14 virion (PDB ID: 8PNF) (67). Images, drawings, and graphs were edited using GIMP and Inkscape software. Statistical analysis was done using OriginPro 2018 software (OriginLab).

## Data Availability

The data that support the findings of this study are available from the authors upon request.
